# Integrated RNA-seq Analysis and Meta-QTLs Mapping Provide Insights into Cold Stress Response in Rice Seedling Roots

**DOI:** 10.3390/ijms21134615

**Published:** 2020-06-29

**Authors:** Weilong Kong, Chenhao Zhang, Yalin Qiang, Hua Zhong, Gangqing Zhao, Yangsheng Li

**Affiliations:** State Key Laboratory of Hybrid Rice, College of Life Sciences, Wuhan University, Wuhan 430072, China; Weilong.Kong@whu.edu.cn (W.K.); zch_nx@whu.edu.cn (C.Z.); qylgzy@whu.edu.cn (Y.Q.); zhonghua0103@whu.edu.cn (H.Z.); zhaogangqing@whu.edu.cn (G.Z.)

**Keywords:** cold stress, *Oryza sativa* (rice), RNA-seq analysis, Meta-QTLs, differentially expressed genes (DEGs)

## Abstract

Rice (*Oryza sativa* L.) is a widely cultivated food crop around the world, especially in Asia. However, rice seedlings often suffer from cold stress, which affects their growth and yield. Here, RNA-seq analysis and Meta-QTLs mapping were performed to understand the molecular mechanisms underlying cold tolerance in the roots of 14-day-old seedlings of rice (RPY geng, cold-tolerant genotype). A total of 4779 of the differentially expressed genes (DEGs) were identified, including 2457 up-regulated and 2322 down-regulated DEGs. The GO, COG, KEEG, and Mapman enrichment results of DEGs revealed that DEGs are mainly involved in carbohydrate transport and metabolism, signal transduction mechanisms (plant hormone signal transduction), biosynthesis, transport and catabolism of secondary metabolites (phenylpropanoid biosynthesis), defense mechanisms, and large enzyme families mechanisms. Notably, the AP2/ERF-ERF, NAC, WRKY, MYB, C2H2, and bHLH transcription factors participated in rice’s cold–stress response and tolerance. On the other hand, we mapped the identified DEGs to 44 published cold–stress-related genes and 41 cold-tolerant Meta-QTLs regions. Of them, 12 DEGs were the published cold–stress-related genes and 418 DEGs fell into the cold-tolerant Meta-QTLs regions. In this study, the identified DEGs and the putative molecular regulatory network can provide insights for understanding the mechanism of cold stress tolerance in rice. In addition, DEGs in KEGG term-enriched terms or cold-tolerant Meta-QTLs will help to secure key candidate genes for further functional studies on the molecular mechanism of cold stress response in rice.

## 1. Introduction

Low temperature is a natural disaster that is often encountered in the entire process of rice growth and development, which is one of the main environmental factors limiting its growth and development, geographical distribution, yield, and quality formation [1,2]. The reduction of rice yield caused by cold stress has been a common problem in the world [2,3,4], especially in China, Japan, Australia, Korea, India, etc. [5,6,7].

The inheritance of cold tolerance in rice is complex and it is difficult to explain the mechanism of cold tolerance with single or several genes [6,7]. According to incomplete statistics, more than 300 QTLs were reported to be involved in cold tolerance using gene mapping studies of various populations at different developmental stages [8]. However, only eight functional cold tolerance genes have been identified by map-based cloning in rice to date, including *qLTG3-1*, *COLD1*, *qCTS-9*, *GSTZ2*, *LTG1*, *Ctb1*, *CTB4a*, and *HAN1* [9,10,11]. Meta-analysis systematically clarifies a phenomenon by fusing a large number of single research results [12]. Meta-QTLs analysis can narrow the QTL confidence interval through math models to further mine candidate genes associated with target traits [8]. Recently, Yang et al. (2018) performed a Meta-QTLs analysis based on 189 public QTLs and obtained 47 cold-tolerant Meta-QTLs (MCqtls) [13]. Besides, many cold response genes that are dependent/independent of the ABA pathway have been cloned in succession, namely, CBFs (Calmodulin-binding transcription activator), MYB transcription factors (TFs), ICE (Inducer of CBF expression) TFs, NAC TFs, etc [14,15,16,17,18,19]. Previous molecular evidence suggested that the sequential expression of *CBFs* and *MYBS3* genes provided two complementary mechanisms for plant cold tolerance. *CBF*’s mediated process initiated immediate cold shock response, while the *MYBS3*-mediated system regulated the long-term cold tolerance of rice [16].

In recent years, RNA-seq technology has the true advantage of detecting the global transcriptome at the whole-genome level and is used to identify cold tolerance genes in rice [20,21]. Wang et al. (2017) identified 121 cold stress-induced genes in cold-tolerant rice and found that a reactive oxygen species (ROS)-bZIP1 regulon plays an important role in early responses to cold stress [22]. Comparative transcriptome analysis of rice LTH (cold tolerant) and IR29 (cold sensitive) identified many stress-inducible genes and emphasized that CBF and MYBS3 regulons are involved in cold tolerance in rice [23]. Similarly, Yang et al. (2015) reported that the cold-tolerant genotype (TNG67) had more rapid alterations in gene expression to tolerate cold stress than the cold-sensitive genotype (TCN1), which related to protein metabolism, modification, folding and defense responses. *OsIAA23*, *SNAC2*, *OsWRKY1v2*, *OsWRKY1v24*, *OsWRKY1v53*, *OsWRKY1v71*, *HMGB*, *OsbHLH*, and *OsMyb* genes may be good candidates for cold stress tolerance in rice [3]. Shen et al. (2014) compared three cold-tolerant genotypes and one cold-sensitive genotype under normal temperature and cold stress treatments and found 318 DEGs as common DEGs related to cold tolerance in the three cold-tolerant genotypes [24]. Maia et al. (2017) compared the transcriptome profiling of rice seedlings between Oro (tolerant) and Tio Taka (sensitive) under cold stress. One hundred and forty-one unique differentially expressed genes (DEGs) were identified in Oro, 5461 were unique DEGs in Tio Taka, and 118 were common DEGs between Oro and Tio Taka [20]. In another study, 13,930 and 10,599 DEGs were detected in the cold susceptible variety (CSV) and cold tolerant variety (CTV) in Pradhan et al.’s study [25]. In weedy rice, Han et al. (2020) identified 2123 up-regulated DEGs and 2522 down-regulated DEGs in a robust cold-tolerant variety (WR157) [26]. Guan et al. (2019) identified 14,213 and 14,730 DEGs in cold-tolerant genotypes (WR 03-35, Kongyu 131), and 9219 and 720 DEGs were obtained in two cold-sensitive genotypes (WR 03-26, 9311) [2].

Although, many rice varieties have been transcriptome analyzed and identified many DEGs. As established, different varieties/genotypes of the same species can also exhibit a high degree of genetic variability in cold tolerance [23,27,28]. Individual unstudied cold-tolerant rice genotype can still identify new key cold-tolerant genes and regulatory networks. On the other hand, previous studies usually obtained massive DEGs, and ignored the use of meta-analysis to reduce the number of candidate DEGs to dig deeper into possible key genes. In this study, the roots of a cold-tolerant rice genotype (RPY geng) seedlings were selected for RNA-seq to analyze the cold stress mechanism. Multiple biological processes and molecular regulation were discussed through various annotation softwares. At last, DGEs were further narrowed down through Meta-QTLs mapping. The study of gene expression profiles in response to cold stress will inspire the regulation network of cold tolerance and provide many candidate genes for agronomic plant manipulation.

## 2. Results

### 2.1. Transcriptome Sequencing and DEGs Analysis

**Figure 1:**
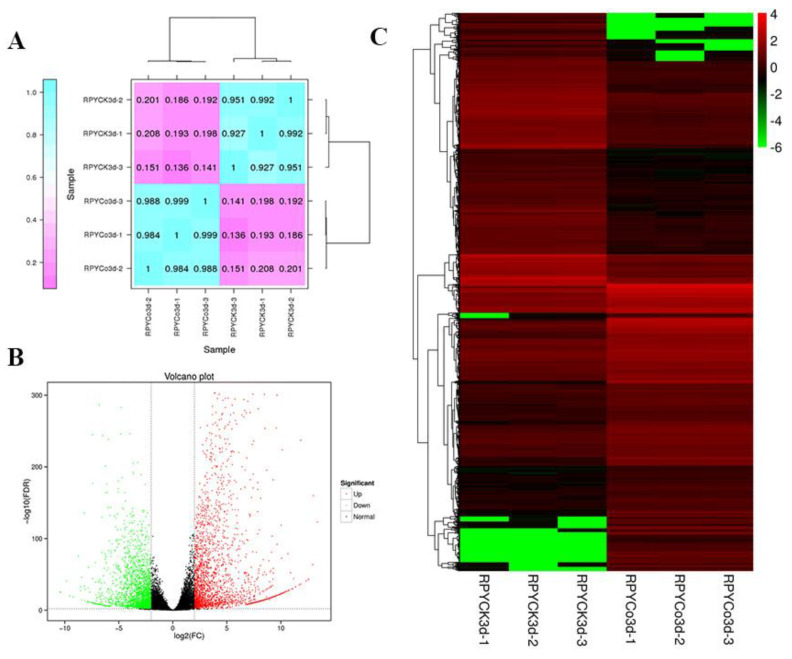
(**A**) Heatmap of Pearson’s correlation between six samples. The value in the heatmap represents the Pearson’s correlation coefficient between the two samples, and the color from pink to blue represents the correlation from weak to strong. (**B**) Volcano Plot of all differentially expressed genes (DEGs). Black dots represent non DEGs, red dots represent upregulated DEGs, and green dots represent downregulated DEGs. (**C**) Heatmap of the expression level of 4779 DEGs. Heatmap is based on FPKM values of all samples. The color from green to red represents the gene expression level from low to high.

A total of 38.29 Gb of clean data was obtained from six samples (Table 1). The percentage of Q30 (an error rate of sequencing lower than 1%_o_) reached over 92.86% and GC contents were between 52.12% and 53.41%. The mapped read ratios of all samples ranged from 78.87% to 83.60%. Pearson’s correlation coefficients of the three biological replicates for each treatment were all greater than 0.92 (Figure 1A). These results indicated that sequencing data can be used in subsequent RNA-seq analysis. According to FDR< 0.01 and |log_2_^fold change^| > 2, we identified 2457 up-regulated and 2322 down-regulated DEGs in response to cold stress (Figure 1B, 1C, Tables S1 and S2). Of them, 1481, 3858, 886, 1874, 4684, 3570, 3213, and 3905 DEGs were annotated in COG, GO, KEGG, KOG, NR, Pfam, Swiss-Prot, and eggNOG databases.

### 2.2. GO, COG, KEGG Enrichment Analysis of the DEGs

**Figure 2:**
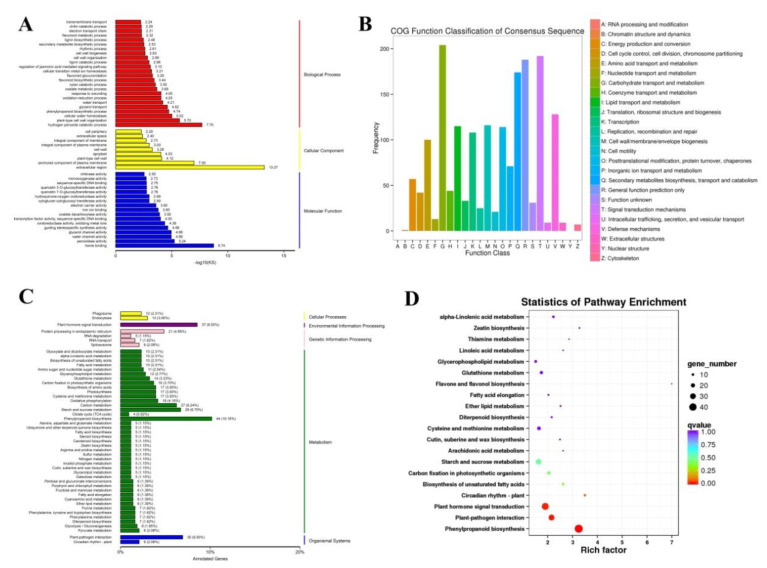
(**A**) Gene ontology (GO) classification of DEGs. Red, yellow, blue terms belong to biological processes, cellular components, and molecular functions, respectively. (**B**) COG function classification of DEGs. Different colored columns represent different functional classifications. (**C**) KEGG classification of DEGs. Different pathways are showed by different columns. The number of DEGs per pathway is quantified by the height of the column. (**D**) KEGG enrichment of DEGs. The number of DEGs is distinguished by the size of the circle and the circle from blue to red represents the q-value from large to small.

The DEGs were assigned to three main categories in the GO enrichment analysis with KS < 0.01: biological processes (29 terms, red terms), cellular components (11 terms, yellow terms), and molecular functions (22 terms, blue terms) (Figure 2A and Table S3). DEGs were significantly enriched in various biological processes (Figure 2A and Table S3), namely, hydrogen peroxide catabolic process (GO:0042744), plant-type cell wall organization (GO:0009664), cellular water homeostasis (GO:0009992), phenylpropanoid biosynthetic process (GO:0009699), glycerol transport (GO:0015793), water transport (GO:0006833), oxidation-reduction process (GO:0055114), and response to wounding (GO:0009611). In molecular functions, DEGs were significantly enriched in heme binding (GO:0020037), peroxidase activity (GO:0004601), water channel activity (GO:0015250), glycerol channel activity (GO:0015254), guiding stereospecific synthesis activity (GO:0042349), as well as oxidoreductase activity, oxidizing metal ions (GO:0016722) (Figure 2A and Table S3). Besides, COG function classification revealed that the DEGs involve several classes: carbohydrate transport and metabolism (204 DEGs), secondary metabolites biosynthesis, transport and catabolism (174 DEGs), signal transduction mechanisms (192 DEGs), and defense mechanisms (128 DEGs) (Figure 2B). On the other hand, the KEGG enrichment analyses of the DEGs highlighted that four KEGG pathways were related to rice cold stress responses/tolerances (Figure 2C,D, and Table S4), namely, phenylpropanoid biosynthesis (ko00940, Figure S1), plant–pathogen interactions (ko04626, Figure S2), plant hormone signal transduction (ko04075, Figure S3), and plant circadian rhythms (ko04712, Figure S4).

### 2.3. Cold Stress-Related TFs and Salt-Stress-Affected Pathways

**Figure 3:**
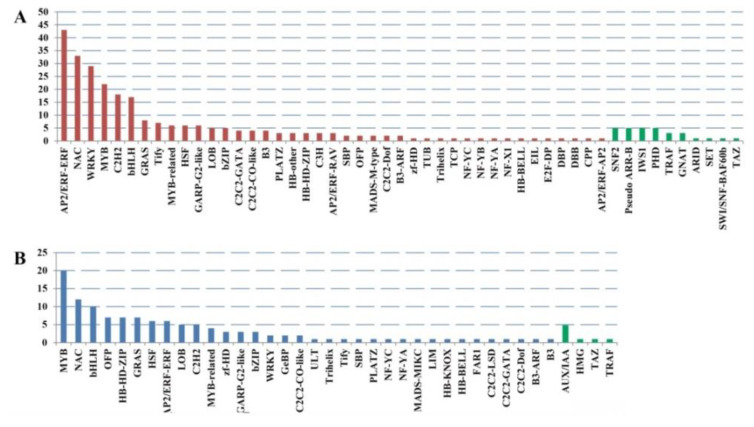
The transcription factors (TFs) and transcriptional regulators (TRs) classification of DEGs by iTAK software. (**A**) The classification results of the TFs and TRs of the up-regulated DEGs. Red bars represent TFs, and green bars represent TRs. (**B**) The classification results of the TFs and TRs of the down-regulated DEGs. Blue bars represent TFs, and green bars represent TRs.

To get cold-responsive TFs in the cold stress regulatory network, various types of TFs and TRs were identified by iTAK software. The top six up-regulated TFs were mainly AP2/ERF-ERF, NAC, WRKY, MYB, C2H2, and bHLH family members (Figure 3). However, the number and types of down-regulated TFs were less than those in up-regulated TFs. The top three down-regulated TFs belonged to MYB, NAC, and bHLH families (Figure 3).

**Figure 4:**
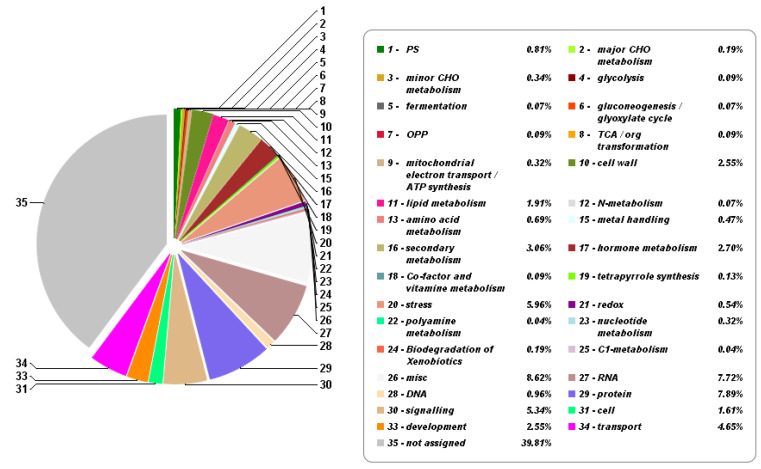
Functional categories of DEGs using MapMan 5.0 with default parameters. The numbers 1–34 in the pie chart represent different patyways. Detailed information on gene numbers and pathway names are also shown in Figure 4. Detailed DEGs annotation results of Table S4 have been provided in Table S5.

**Figure 5:**
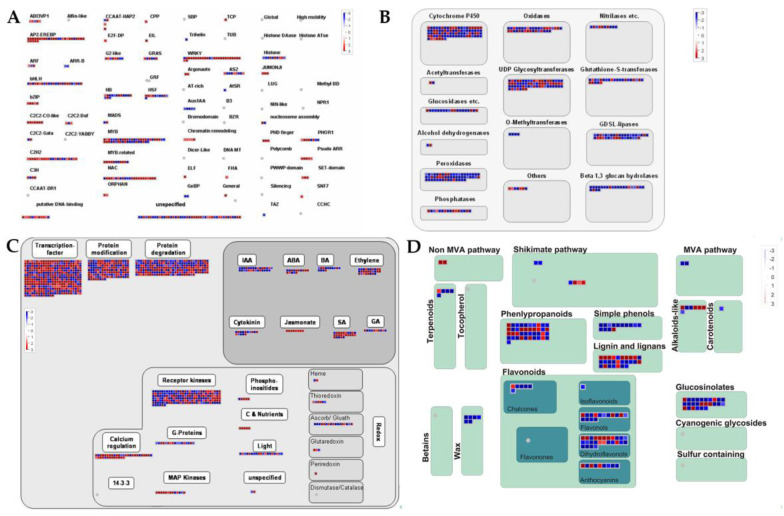
MapMan overview of changes in the expression of DEGs involved in transcription factor regulation (**A**), large enzyme families (**B**), plant growth regulation containing protein modification, protein degradation, plant hormones, and redox process (**C**), and the secondary metabolisms (**D**). Each square in A–D represents a separate DEG, red indicates that gene expression was induced and blue indicates that gene expression was repressed compared with the control.

We mapped DEGs to different functional categories in the MapMan tool to reveal the salt-stress-affected pathways. All DEGs were associate to 35 pathways (Figure 4 and Table S5). They were significantly enriched on misc (8.62%), stress (5.96%), signaling (5.34%), secondary metabolism (3.06%), and hormone metabolism (2.70%) except for RNA (7.72%), protein (7.89%), and transport (4.65%) (Figure 4). Therefore, these process genes were listed in detail in Figure 5. Mapman’s identification of TFs was similar to that of iTAK software. Most AP2-EREBP, WRKY, and C2H2 TFs genes were up-regulated, while half of the bHLH and MYB TFs genes were down-regulated (Figure 5A). Interestingly, four bZIP TFs genes showed obvious up-regulation under cold stress (Figure 5A). A large number of large enzyme families are also differentially expressed under cold stress (Figure 5B). This result indicated that cold stress severely affected the enzyme homeostasis in the rice roots. Multiple IAA, ABA, BA, Ethylene, Cytokinin, Jasmonate, SA, and GA pathways genes were DEGs (Figure 5C), which also fully supported the KEGG enrichment result (ko04075, Figure S3). Many DEGs were related to protein modification and protein degradation (Figure 5C). As expected, redox and signaling DEGs including G-proteins, MAP Kinases, Calcium regulation, etc., were found (Figure 5C). In addition, we noticed a lot of secondary metabolism-related DEGs (Figure 5D). These results proved that rice cold stress tolerance was a very complex regulatory process involving multiple pathways and transcription factors.

### 2.4. Key DEGs locking Involved in the Response to Cold Stress

**Figure 6:**
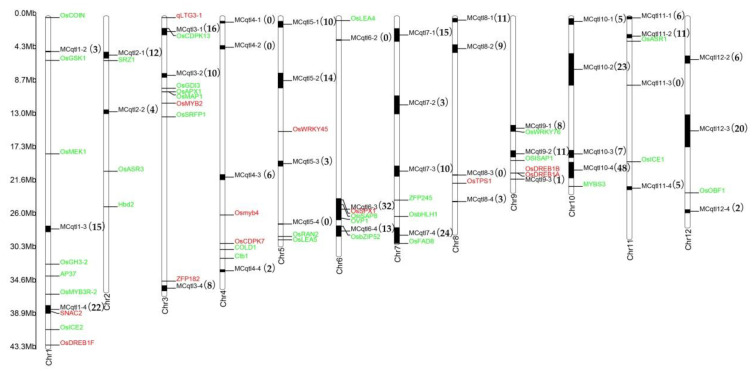
DEGs mapping in Meta-QTLs from Yang et al. (2018). The green genes were cold–stress-related genes that have been cloned previously, but not the DEGs of this study. The red genes were the DEGs in this study and also belong to cold–stress-related genes previously reported. Black boxes in each chromosome (Chr) represent Meta-QTLs (e.g., MCqtl1-2, MCqtl1-3) and the number after Meta-QTL represents the number of our DEGs in this Meta-QTL segment.

Cold stress has a great influence on the growth and yield of rice, therefore, for many years, a large number of cold stress functional genes have been cloned and demonstrated through different rice materials, genetic population, and cloning methods, such as *COLD1* [10], *OsCDPK7* [29], *OsWRKY45* [30], etc. We found that 12 DEGs in this study are genes that have been cloned (Table 2 and Figure 6). A total of 417 DEGs were dropped into 41 Meta-QTLs (Figure 6, Table S6, and S7). Interestingly, *EVM0001397* (*SNAC2*) and *EVM0031981* (*OsSPX1*) were in MCqtl1-4 and MCqtl6-3, respectively. This result indicated that DEGs analysis combined with Meta-QTLs mapping can effectively reduce the number of candidate genes and accurately locate the key genes of cold stress. Forty-one Meta-QTLs contained 0 to 48 DEGs, and the number of DEGs was much smaller than the number of genes in Meta-QTLs. Of them, the number of DEGs in 36 Meta-QTLs was less than 20 (Figure 6, Tables S6 and S7), especially, MCqtl9-3 (1 DEGs), MCqtl4-4 (2 DEGs), MCqtl12-4 (2 DEGs), MCqtl1-2 (3 DEGs), MCqtl7-2 (3 DEGs), MCqtl8-4 (3 DEGs), MCqtl2-2 (4 DEGs), MCqtl5-3 (4 DEGs), MCqtl10-1 (5 DEGs), and MCqtl11-4 (5 DEGs). *EVM0009341* was the only DEGs in MCqtl9-3, annotated as Myb-related protein. This gene and the previously cloned cold-stress corresponding genes (*Osmyb4* [31], *MYBS3* [16], and *OsMYB3R-2* [32,33]) belonged to the same family and showed a 3.63-fold upregulation (Figure 6 and Table S7). MCqtl4-4 included *EVM0032414* related to amino acid transport and metabolism and *EVM0018896* (Function unknown). Two DEGs in MCqtl12-4 were annotated as Auxin-responsive protein IAA30 (*EVM0025632*, KEGG: ko04075) and CBL-interacting protein kinase 4 (*EVM0013862*). MCqtl1-2 also had a DEG in signal transduction mechanisms. Similarly, other Meta-QTLs also contained one or several potential cold-stress-responsive DEGs.

## 3. Discussion

### 3.1. Cold-Stress-Responsive DEGs in Rice Seedling Roots Associated with Plant Stress/Hormone Signal Transduction, Secondary Metabolites Mechanisms, TFs, and Function Proteins

Cold stress tolerance is a complicated quantitative trait that is controlled by various quantitative trait loci [3,15]. RNA-seq technology can identify a large number of cold-stress-responsive DEGs [24,34]. The obtained DEGs were systematically classified by various enrichment methods such as GO, COG, KEGG, and Mapman, which can fully analyze the cold stress regulation mechanism in rice roots. In this study, we obtained 4779 DEG-associated multiple biological processes. Lots of DEGs involved redox regulation, peroxidases, alcohol dehydrogenases, and glutathione S transferases, which implied that rice roots have suffered a certain degree of damage under cold stress. Previous studies proposed that cross-talk of plant hormones play important roles in the plant responses and resistance to multiple abiotic stresses [3,26]. Different plant hormones usually perform different functions, but their integration or coordinated expression is very important for plant growth and even survival under multiple stress environments [35,36]. Our KEGG enrichment term (ko04075, Figure 2C,D) and Mapman enrichment (Figure 5C) revealed that most of plant hormones DEGs including IAA, ABA, BA, Ethylene, Cytokinin, Jasmonate (JA), SA, and GA, participated in the cold stress response of rice roots. In addition, DEGs in Ca^2+^ signal transduction and MAPK cascade pathway were differently expressed (Figure 5C), which supported earlier discovery that Ca^2+^ signal transduction and MAPK cascade pathways were two major categories mediating stress signal transduction [24,25,34]. Interestingly, the expression levels of all JA DEGs were up-regulated. In *Arabidopsis thaliana*, JA enhanced the freezing tolerance by positively regulating the ICE-CBF/DREB1 pathway [37]. Therefore, we speculated that rice also enhances cold stress tolerance through this pathway. Wu et al. (2014) showed that cold acclimation is related to disease resistance in Amur grape (*Vitis amurensis*) [38]. Our DEGs were significantly enriched in plant–pathogen interactions (ko04626, Figure S2) and 34 pathogenesis-related proteins (PR-proteins) DEGs were found. These results suggested that the plant–pathogen interaction pathway may also play a role in cold resistance.

**Figure 7:**
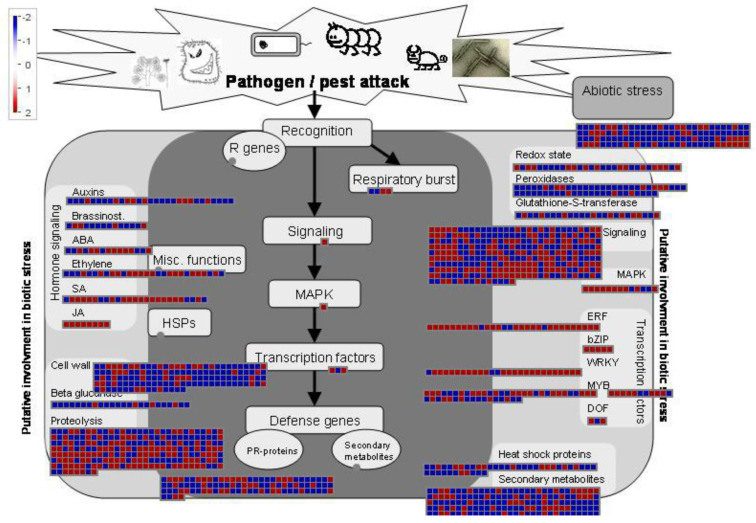
The model of the regulation mechanism of rice seedling root response to cold stress involves numerous pathways. DEGs involving these pathways are represented by small squares. The color of the small square reflects the change of DEGs after cold treatment at 5 °C. Red/blue indicates that gene expression was induced/repressed compared with the control. Detailed information about all DEGs and pathways can be obtained in Table S5.

On the other hand, KEGG, GO enrichment, and COG function classification all pointed out that secondary metabolites play an important role in rice cold tolerance, especially phenylpropanoid biosynthesis (GO: 0009699, ko00940). Phenylpropanoids play a key role in the growth and development of plants and response to stress [39,40]. Our analysis result of DEGs showed that many secondary metabolites were widely involved in cold stress tolerance in rice. Earlier studies cloned some TFs that can enhance rice cold stress tolerance, namely, OsMYB2 [41], OsWRKY45 [30], Osmyb4 [42], etc. According to TF’s identification result from iTAK and Mapman software, various *AP2/ERF-ERF*, *NAC*, *WRKY*, *MYB*, *C2H2*, and *bHLH* TF genes were up-/down-regulated. These DEGs, especially those that fall on Meta-QTLs, may be good candidates for transgenic genes to enhance cold stress tolerance in rice. Finally, all regulatory DEGs were summarized and presented through Mapman (Figure 7).

### 3.2. Meta-QTLs Mapping of DEGs Can Effectively Lock Main Effect Candidate Genes Related to Cold Stress Tolerance in Rice

Hu et al. (2008) reported that rice *SNAC2* conferred cold and salt tolerance in rice [43]. Increased expression of *OsSPX1* caused high sensitivity to cold and oxidative stresses in rice seedlings [44] and enhanced cold/subfreezing tolerance in tobacco and *A. thaliana* [45]. Yang et al. (2018) effectively reduced the physical distance MCqtl1-4 and MCqtl6-3 to 1.0 and 2.7 Mb based on Meta-QTLs analysis, respectively. However, most of the Meta-QTLs still contained many genes. Fortunately, we found that the possible genes in Meta-QTLs were greatly reduced after DEGs mapping of their Meta-QTLs. It should be noted that two previously cloned genes, *SNAC2* and *OsSPX1*, were highlighted in MCqtl1-4 and MCqtl6-3 after our DEGs Meta-QTLs mapping. This result indicated that DEGs Meta-QTLs mapping is an effective method to discover the major genes in Meta-QTLs regarding cold stress tolerance. As expected, in Meta-QTLs containing very few DEGs, we have found possible candidate genes and these genes were supported by multiple annotation results. For example, *EVM0013862* encoded CBL-interacting protein kinase 4 belonging to Ca^2+^ signal transduction pathway in MCqtl12-4 (only two DEGs) and *EVM0009341* encoding Myb-related protein was only a DEGs in MCqtl9-3. In this study, DEGs Meta-QTLs mapping made all 28 Meta-QTLs containing less than 15 genes (Tables S6 and S7).

However, MCqtl3-1, MCqtl12-3, MCqtl1-4, MCqtl10-2, MCqtl6-3, MCqtl7-4, and MCqtl10-4 contained 16–48 DEGs. Therefore, we provided comparison results of multiple databases to facilitate the further screening of candidate genes through annotations (Table S7). Of course, our screened genes in Meta-QTLs can be further fine mapped by developing SNP markers in flanking of these genes in the previously reported population, observing the mutant phenotypes of these genes from the published mutant library, or building CRISPR/Cas9 plants for cold stress experiments.

## 4. Materials and Methods

### 4.1. Plant Materials and Treatments

Fourteen-day-old seedlings with uniform growth of rice cultivar ‘RPY geng’ (*Oryza sativa* ssp. *japonica*) were grown in 96-well PCR plate as supporting materials in Yoshida solution (Coolaber, Beijing, China) and changed to 5 °C (cold treatment)/26 °C (control), with a 16/8 h light/dark photoperiod, 60% relative humidity in plant growth incubators (ZSX1500GS, Jingshen Instrument, Shanghai, China) for three days, respectively. Here, three biological replicates were set using three separate cold/control treatments [20,46]. Only 1–2 roots from each seedling were collected immediately with liquid nitrogen for RNA extraction in order to include maximum number of plants (> 30 rice seedling) in single biological replicate [46,47].

### 4.2. Transcriptome Sequencing and RNA-seq Analysis

RNA extraction and cDNA library construction of the total samples were conducted by Biomarker Technologies (Beijing, China) as described previously [48,49,50]. Then, these cDNA libraries were sequenced on an Illumina sequencing platform (HiSeq 2500) [48,50]. All raw reads after sequencing were filtered using Trimmomatic and raw reads-containing adapter, ploy-N, and low-quality reads were removed. The clean data with high quality were used for the RNA-seq analyses. Q30, GC-content, and sequence duplication level of the clean data were calculated, respectively. HISAT2 software was used to map all clean reads to the reference genome of RPY geng (unpublished data) [51,52], and the mapped reads were assembled and quantified using StringTie software [53]. Gene expression levels were estimated by fragments per kilobase of transcript per million fragments mapped reads (FPKM) method [54,55]. Pearson’s correlation coefficient of all tested samples was calculated based on all FPKMs. DESeq2 was used to identify differential expression genes (DEGs) with a False Discovery Rate (FDR) < 0.01 and |log_2_^fold change^| > 2 [34].

### 4.3. Functional Annotation and GO, KEGG Enrichment Analysis of the DEGs

Gene functions of DEGs were annotated by BlastP the following databases (E-value of e^−5^): Nr (NCBI non-redundant protein sequences); Nt (NCBI non-redundant nucleotide sequences); Pfam (Protein family); KOG/COG (Clusters of Orthologous Groups of proteins); Swiss-Prot (A manually annotated and reviewed protein sequence database); KO (KEGG Ortholog database); GO (Gene Ontology).

Gene Ontology (GO) enrichment analysis of the DEGs was implemented by the GOseq R packages based Wallenius non-central hyper-geometric distribution [56], which can adjust for gene length bias in DEGs. GO terms of DEGs with KS < 0.01 were considered significantly enriched. We used KOBAS software to test the statistical enrichment of differential expression genes in KEGG pathways [57]. KEGG terms of DEGs with corrected P-value < 0.05 were considered significantly enriched [26].

The full-length nucleotide sequences of DEGs were submitted to the online annotation tool of Mapman (http://www.plabipd.de/portal/mercator-sequence-annotation) for functional annotation using default parameters [58]. Mapman annotations and gene expression levels of DEGs were visualized by Mapman [58].

### 4.4. Transcription Factors (TFs) and Transcriptional Regulators (TRs) Identification and Meta-QTLs Mapping.

We used iTAK software (http://itak.feilab.net/cgi-bin/itak/index.cgi) to identify TFs and TRs of DEGs with default parameters [59]. According to statistics from the National Rice Data Center (http://www.ricedata.cn/gene/) and the published papers, 44 cold-stress-related genes have been cloned. To determine how many of our DEGs are cloned genes, gene IDs of IRGSP-1.0 and nucleotide sequences of these genes were downloaded. Orthologs of our DEGs matched to genes in IRGSP-1.0 by blastn search using the following parameters: max_target_seqs 1, evalue e^−5^, perc_identity 95 [34]. Then, DEGs matching the cloned genes were listed.

Meta-QTLs were obtained from Yang et al. study [13]. The physical location in IRGSP-1.0 of Meta-QTLs determined by online blast tool (https://rapdb.dna.affrc.go.jp/tools/blast) in the RAP-DB website (https://rapdb.dna.affrc.go.jp/download/irgsp1.html); the sequence of SSR markers must be identical with that of the whole genome sequence (100% identical, unique location). Then, the DEGs falling into the physical interval of Meta-QTLs were summarized, including our DEGs’ ID, IRGSP-1.0, FPKM, function annotations, and other information.

## 5. Conclusions

In this study, we have identified numerous DEGs involving the putative molecular regulatory network of rice cold stress tolerance through the systematic RNA-seq analysis of the roots of fourteen-day rice seedlings. Our DEGs mapping to cold-tolerant Meta-QTLs previously reported provides effective target genes for future cold stress resistant rice breeding.

## Figures and Tables

**Table 1:** Summary information of sequencing data and mapped ratios.

Samples	Total Reads	GC Content	% ≥ Q30	Mapped Reads
RPYCK3d-1	42,173,470	53.07%	93.34%	34,945,260 (82.86%)
RPYCK3d-2	42,574,242	52.95%	93.55%	35,593,632 (83.60%)
RPYCK3d-3	40,767,720	53.41%	94.41%	33,216,467 (81.48%)
RPYCo3d-1	45,230,168	52.24%	93.31%	36,277,084 (80.21%)
RPYCo3d-2	42,541,038	52.12%	93.27%	33,553,268 (78.87%)
RPYCo3d-3	42,812,246	52.36%	92.86%	34,405,870 (80.36%)

**Table 2:** Our DEGs in cold stress tolerance genes previously cloned.

Gene IDs	Published Genes	FDR	Log_2_^FC^	Regulated
*EVM0041152*	*OsCDPK7*	1.48E-188	3.005073	up
*EVM0023819*	*OsDREB1B*	9.80E-161	12.99874	up
*EVM0037161*	*OsDREB1F*	2.69E-14	8.265282	up
*EVM0000436*	*OsMYB2*	0	7.485158	up
*EVM0001849*	*OsDREB1A*	0	9.69025	up
*EVM0001397*	*SNAC2*	0	7.397628	up
*EVM0005553*	*OsWRKY45*	1.28E-239	5.073813	up
*EVM0042736*	*OsTPS1*	1.18E-189	4.569165	up
*EVM0031981*	*OsSPX1*	1.48E-128	3.062169	up
*EVM0041750*	*ZFP182*	0	8.455205	up
*EVM0018891*	*Osmyb4*	0	5.400831	up
*EVM0018509*	*qLTG3-1*	8.43E-43	-4.27137	down

Note: FDR means false discovery rate and is obtained by the Benjamini–Hochberg correction method to correct the p-value of significance difference.

## Supplementary Materials

The following are available online at https://www.mdpi.com/1422-0067/21/13/4615/s1, Table S1: Statistics and details of DEGs in this study; Table S2: Full-length nucleotide sequences of all DEGs; Table S3: The topGO result of DEGs; Table S4: The KEGG statistical result of DEGs; Table S5: Mapman enrichment result of DEGs; Table S6: Detailed information of Meta-QTLs; Table S7: Detailed information of DEGs in Meta-QTLs. Figure S1: KEGG term of phenylpropanoid biosynthesis (ko00940); Figure S2: KEGG term of plant hormone signal transduction (ko04075); Figure S3: KEGG term of plant-pathogen interaction (ko04626); Figure S4: KEGG term of circadian rhythm—plant (ko04712).
