# Supplementary figures and images for: Integrated RNA-seq Analysis and Meta-QTLs Mapping Provide Insights into Cold Stress Response in Rice Seedling Roots
Source: Int J Mol Sci. 2020 Jun 29;21(13):4615. doi: 10.3390/ijms21134615 (PMC7369714; doi:10.3390/ijms21134615)

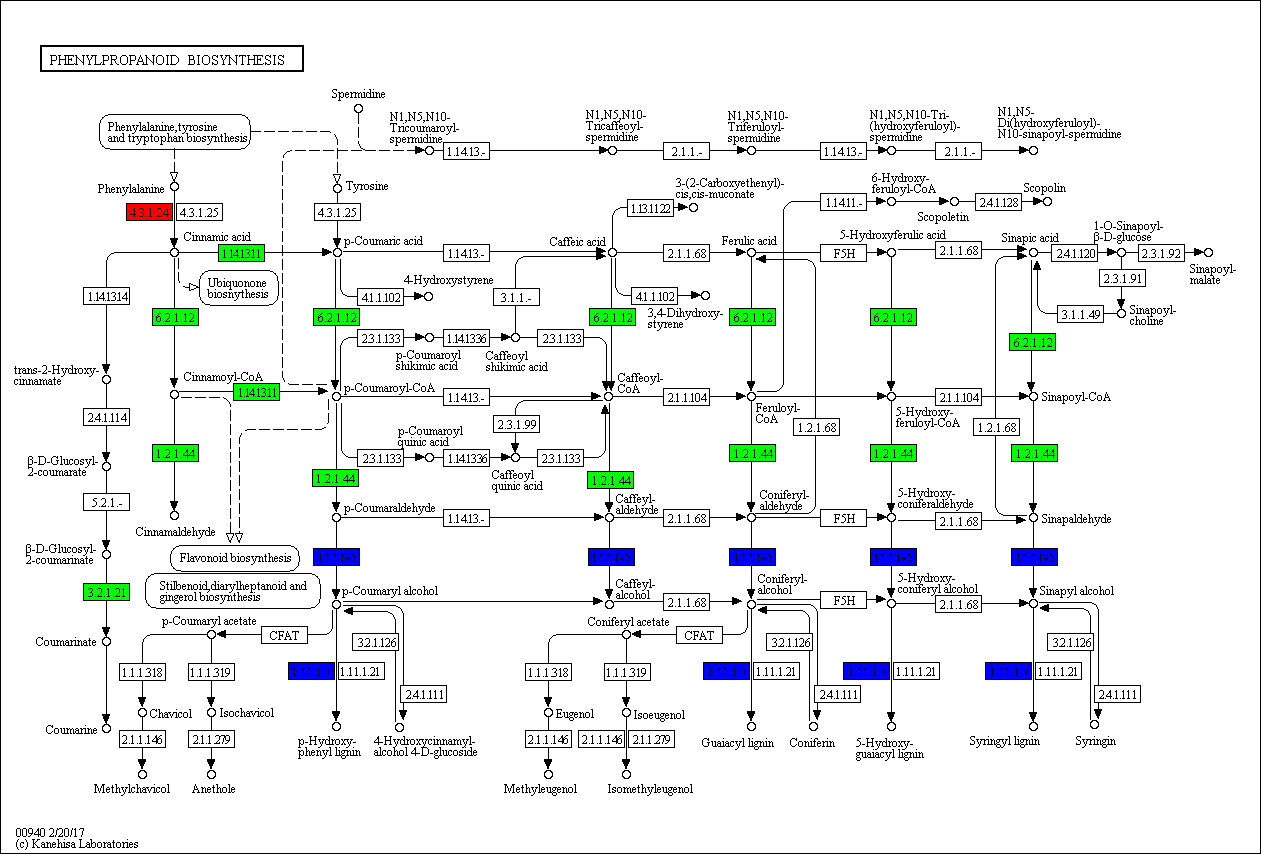

Supplement: Supplementary file 1 [file ijms-21-04615-s001.zip › Supplementary/Figure S1.png]

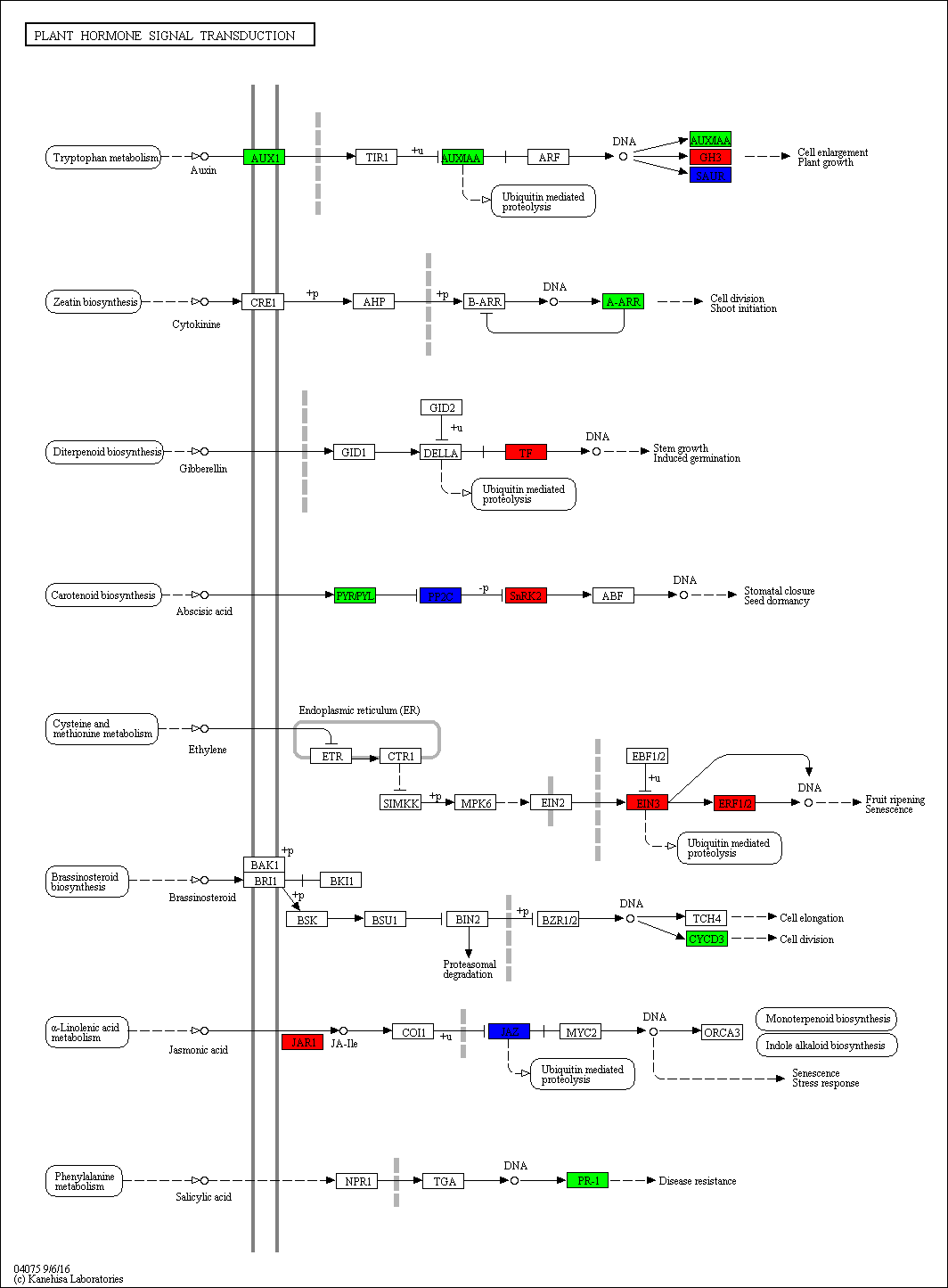

Supplement: Supplementary file 1 [file ijms-21-04615-s001.zip › Supplementary/Figure S2.png]

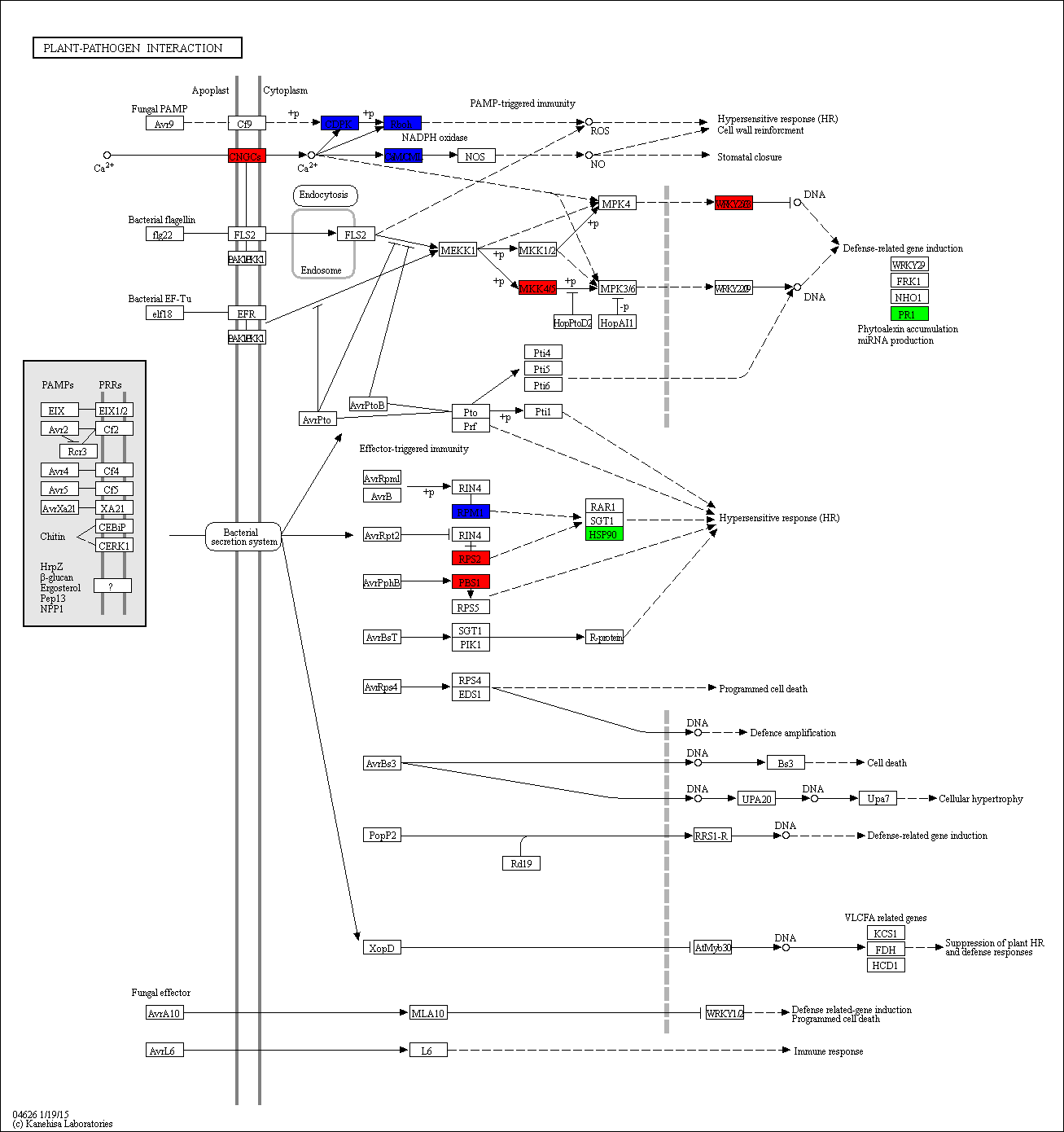

Supplement: Supplementary file 1 [file ijms-21-04615-s001.zip › Supplementary/Figure S3.png]

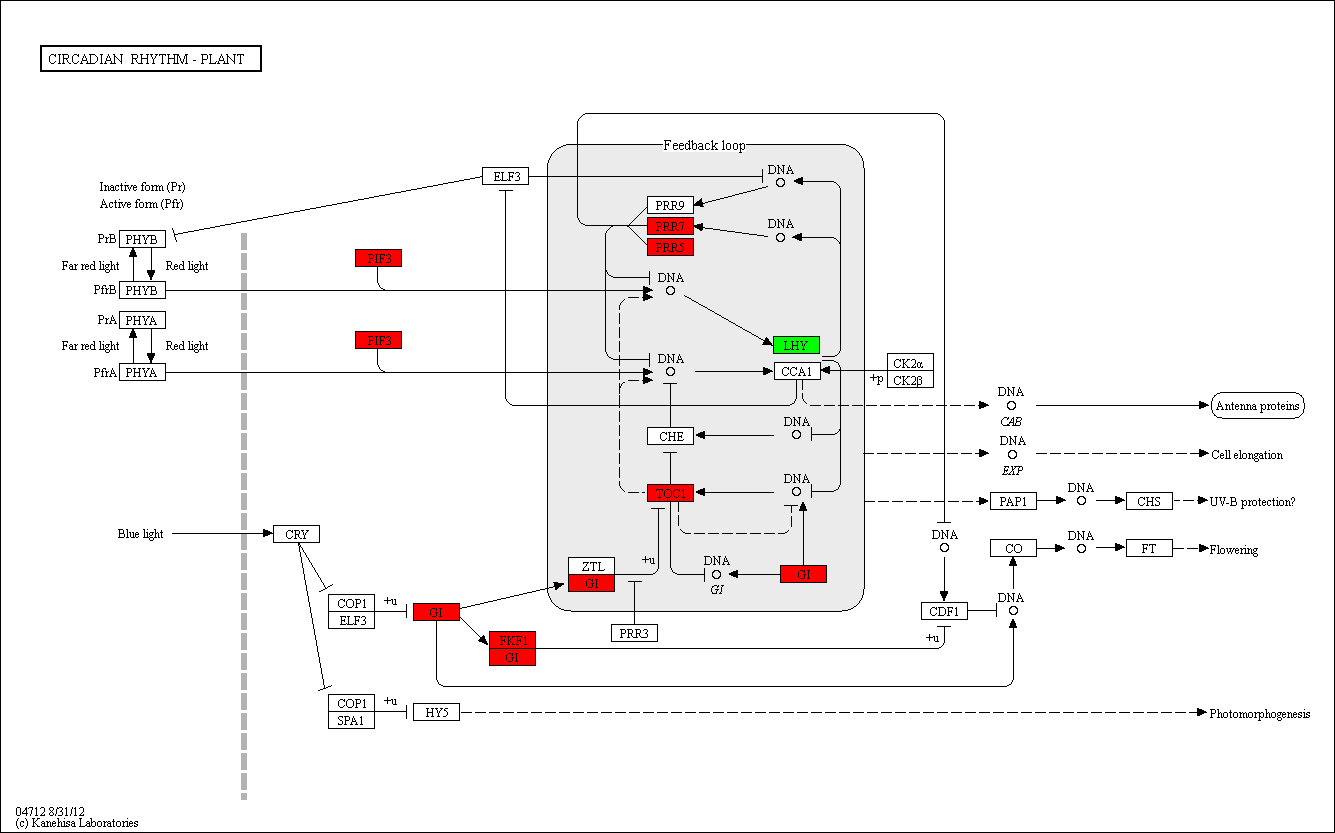

Supplement: Supplementary file 1 [file ijms-21-04615-s001.zip › Supplementary/Figure S4.png]
